# Artificial intelligence based system for predicting permanent stoma after sphincter saving operations

**DOI:** 10.1038/s41598-023-43211-w

**Published:** 2023-09-25

**Authors:** Chih-Yu Kuo, Li-Jen Kuo, Yen‑Kuang Lin

**Affiliations:** 1 Department of Surgery, Taipei Medical University Hospital, Taipei, Taiwan; 2 Division of Colorectal Surgery, Department of Surgery, Taipei Medical University Hospital, Taipei Medical University, Taipei, Taiwan; 3 Department of Surgery, School of Medicine, College of Medicine, Taipei Medical University, Taipei, Taiwan; 4 Taipei Cancer Center, Taipei Medical University, Taipei, Taiwan; 5 Graduate Institute of Athletics and Coaching Science, National Taiwan Sport University, Taoyuan, Taiwan

**Keywords:** Gastroenterology, Medical research, Oncology

## Abstract

Although the goal of rectal cancer treatment is to restore gastrointestinal continuity, some patients with rectal cancer develop a permanent stoma (PS) after sphincter-saving operations. Although many studies have identified the risk factors and causes of PS, few have precisely predicted the probability of PS formation before surgery. To validate whether an artificial intelligence model can accurately predict PS formation in patients with rectal cancer after sphincter-saving operations. Patients with rectal cancer who underwent a sphincter-saving operation at Taipei Medical University Hospital between January 1, 2012, and December 31, 2021, were retrospectively included in this study. A machine learning technique was used to predict whether a PS would form after a sphincter-saving operation. We included 19 routinely available preoperative variables in the artificial intelligence analysis. To evaluate the efficiency of the model, 6 performance metrics were utilized: accuracy, sensitivity, specificity, positive predictive value, negative predictive value, and area under the receiving operating characteristic curve. In our classification pipeline, the data were randomly divided into a training set (80% of the data) and a validation set (20% of the data). The artificial intelligence models were trained using the training dataset, and their performance was evaluated using the validation dataset. Synthetic minority oversampling was used to solve the data imbalance. A total of 428 patients were included, and the PS rate was 13.6% (58/428) in the training set. The logistic regression (LR), Gaussian Naïve Bayes (GNB), Extreme Gradient Boosting (XGB), Gradient Boosting (GB), random forest, decision tree and light gradient boosting machine (LightGBM) algorithms were employed. The accuracies of the logistic regression (LR), Gaussian Naïve Bayes (GNB), Extreme Gradient Boosting (XGB), Gradient Boosting (GB), random forest (RF), decision tree (DT) and light gradient boosting machine (LightGBM) models were 70%, 76%, 89%, 93%, 95%, 79% and 93%, respectively. The area under the receiving operating characteristic curve values were 0.79 for the LR model, 0.84 for the GNB, 0.95 for the XGB, 0.95 for the GB, 0.99 for the RF model, 0.79 for the DT model and 0.98 for the LightGBM model. The key predictors that were identified were the distance of the lesion from the anal verge, clinical N stage, age, sex, American Society of Anesthesiologists score, and preoperative albumin and carcinoembryonic antigen levels**.** Integration of artificial intelligence with available preoperative data can potentially predict stoma outcomes after sphincter-saving operations. Our model exhibited excellent predictive ability and can improve the process of obtaining informed consent.

## Introduction

Artificial intelligence (AI), which refers to the ability of machines to mimic human cognitive functions and achieve particular goals by using input data^1^, has become prevalent in most fields. AI is widely applied in medical research and health care for, for example, diagnostic imaging analysis, pathological interpretation, disease prognosis prediction, complication prevention, skills training, and assessment^2^. In the present study, we used AI to build a model to precisely predict the probability of a permanent stoma (PS) forming before patients with rectal cancer underwent a sphincter-saving operation.

Obtaining surgical informed consent is a crucial component of modern medicine that enhances patient compliance and satisfaction^3^. However, in rectal cancer treatment, the process of obtaining surgical informed consent is frequently inadequate^4^. Many patients with rectal cancer refuse therapy or opt out of surgery because they fear developing a PS. In addition, a misunderstanding of and unrealistic expectations for surgery may reduce patient satisfaction and lead to legal disputes. Although the goal of rectal cancer treatment is to restore gastrointestinal continuity, 3–25% of patients with rectal cancer experience a PS after a sphincter-saving operation^5,6^. Therefore, surgeons must provide information regarding the possibility of a PS forming when they obtain informed consent before surgery. Although many studies have identified risk factors for PS, few studies have precisely predicted the probability of a PS forming before surgery.

Although PS formation is a multifactorial and complex problem, deep learning, a mode of AI, can be used to construct models that learn logical patterns from a large amount of routinely available preoperative data to predict the probability of PS formation. In the current study, we developed an individualized model for the accurate prediction of PS formation. This AI model can be integrated into the process of obtaining informed consent before a sphincter-saving operation.

## Materials and methods

### Patient selection and follow-up

We obtained the data of 428 patients who underwent a sphincter-saving operation for rectal cancer between January 1, 2012, and December 31, 2021, at Taipei Medical University Hospital. The malignancy of the rectum had been confirmed through biopsy for all patients, and endoscopic findings revealed that all lesions had a distal border within 12 cm of the anal verge. We excluded patients who had stage IV disease and had undergone emergency surgery or abdominoperineal resection (APR). After the operation, the patients were followed up at 3-month intervals for the first 2 years and then at 6-month intervals until the end of 5 years. After 5 years of follow-up, we followed these patients annually until their death or loss to follow-up. All patients were followed at least 1 year. A patient was considered to have a PS if their stoma was still present at the end of the follow-up period (post-operative follow till December 31, 2022). This study was performed in accordance with the guidelines of the Declaration of Helsinki. This study was approved by the Joint Institutional Review Board of Taipei Medical University (TMU-JIRB No.: N202212085), and the review board waived the requirement to obtain the informed consent.

### Preoperative clinical variables

The following 19 preoperative variables were included in the AI analysis: age, sex, body mass index (BMI), comorbidities (diabetes mellitus [DM], hypertension, heart disease, chronic obstructive pulmonary disease [COPD], chronic kidney disease [CKD], and liver disease), smoking status, the distance of the lesion from the anal verge, whether receiving neoadjuvant concurrent chemoradiotherapy (CCRT), American Society of Anesthesiologists (ASA) score, preoperative laboratory data (hemoglobin [Hb], albumin, and carcinoembryonic antigen [CEA]), clinical T stage, clinical N stage and clinical stage. All data were obtained from medical records and imaging findings obtained a few days before operation. The distance from the lesion to the anal verge was measured during preoperative colonoscopy. The clinical stage was confirmed on the basis of the findings of total-body contrast-enhanced computed tomography and magnetic resonance imaging of the pelvis.

### Statistical analysis

The target variable of this study was the development of a PS after a sphincter-saving operation, and this variable was evaluated on the basis of both demographic and clinical data. Identifying where a patient will develop a PS is a typical classification task in machine learning. We employed the following machine learning models: logistic regression (LR), random forest (RF), decision tree (DT), Gaussian Naïve Bayes (GNB), extreme gradient boosting (XGB), gradient boosting (GB), and light gradient boosting machine (LGBM). We analyzed the performance of each model and compared the features selected by these 7 models. In our classification pipeline, the data were randomly divided into training set (80% of the data) and a validation set (20% of the data). The models were trained using the training dataset. The performance of the models was evaluated using the validation dataset. The 7 models used in this study are described in the following.

LR is commonly used to solve binary classification problems in which the goal is to predict the probability of an event occurring (e.g., whether a customer will make a purchase) on the basis of a set of features or predictors (e.g., demographic information and purchase history).

RF is a machine learning algorithm widely used to perform both classification and regression tasks. RF is an ensemble learning method that involves constructing a collection of DTs during training and identifying the class that represents the mode of the classes (for classification) or the mean prediction (for regression) of individual trees. An advantage of RF is its ability to reduce overfitting, which can occur in a single DT.

The DT provides a graphical representation of a series of decisions or actions, with each decision or action leading to 1 or more possible outcomes. The tree is composed of nodes, which represent the decisions or actions, and edges, which connect the nodes and represent the possible outcomes. In a DT, the root node represents the starting point, and each subsequent node represents a decision or action that can be taken on the basis of available information. At each node, the decision is made on the basis of a set of conditions or rules, and the outcome determines the branch of the tree to be followed. The final nodes of the tree are called leaf nodes and represent the predicted outcome or decision.

GNB is a simple probabilistic algorithm used in machine learning to perform classification tasks. GNB is based on Bayes’ theorem, which is used to calculate the probability of a class with some observed features. GNB assumes that the features of each class are normally distributed, with a mean and variance that are specific to that class. For a new set of features, GNB can calculate the probability of each class by using Bayes’ theorem and select the class with the highest predicted probability.

XGB is a widely used machine learning algorithm that falls under the category of boosting algorithms. It is a highly scalable, efficient, and accurate algorithm used for regression, classification, and ranking tasks. XGB sequentially builds an ensemble of DTs, with each tree correcting errors made by the previous tree. During the training phase, XGB optimizes an objective function that measures the difference between predicted and actual values. XGB uses GB and regularization techniques to improve the performance of the algorithm and prevent overfitting.

GB involves the construction of a series of simple prediction models, such as DTs, that are combined in an additive manner to produce a final prediction model. GB sequentially fits new models to the residuals (i.e., the errors) of previous models. Each new model is trained to predict the negative gradient of the loss function with respect to current predictions. With the addition of these new models to the ensemble, the overall error of the model is gradually reduced.

LGBM is a machine learning algorithm used to perform classification, regression, and ranking tasks. It is based on a GB framework and uses a histogram-based approach to speed up training and reduce memory consumption.

To evaluate the efficiency of the models, we used 6 performance metrics: accuracy, sensitivity, specificity, positive predictive value, negative predictive value, and area under the receiver operating characteristics curve (AUROC). Accuracy was determined by dividing the sum of true positive and true negative predictions by the total number of positive and negative samples. Sensitivity refers to the true positive rate, which is the proportion of actual positives that are correctly identified by a binary classification model. Sensitivity is used to measure how efficiently a model can detect positive cases when they are present. Specificity refers to the ability of a test or measure to correctly identify individuals who do not have a particular condition or characteristic. AUROC is a measure of the performance of a binary classification model. The receiver operating characteristic (ROC) curve is a plot of the true positive rate against the false positive rate for different threshold values of a model’s predicted probability. The AUROC is a measure of how efficiently the model can distinguish between the 2 classes. An AUROC score of 1.0 indicates perfect discrimination between positive and negative classes, whereas a score of 0.5 indicates random guessing. The AUROC is a commonly used metric for binary classification problems, especially in the contexts of medical research and machine learning. This metric is preferred over other metrics, such as accuracy, when classes are imbalanced or when the cost of false positives and false negatives is unequal.

In the present study, the data imbalance was solved using the synthetic minority oversampling technique (SMOTE). SMOTE is a data augmentation technique commonly used in machine learning to address class imbalance problems in a classification task. Imbalance can cause a machine learning model to be biased toward the majority class and exhibit poor predictive performance for the minority class. SMOTE creates synthetic samples from the minority class by interpolating between existing samples. SMOTE selects a minority sample and identifies its k-nearest neighbors. Subsequently, it creates synthetic samples along line segments connecting the selected sample to each of its k-nearest neighbors. This generates synthetic minority samples that are similar to the existing minority samples.

## Results

### Patient characteristics

**Figure 1:**
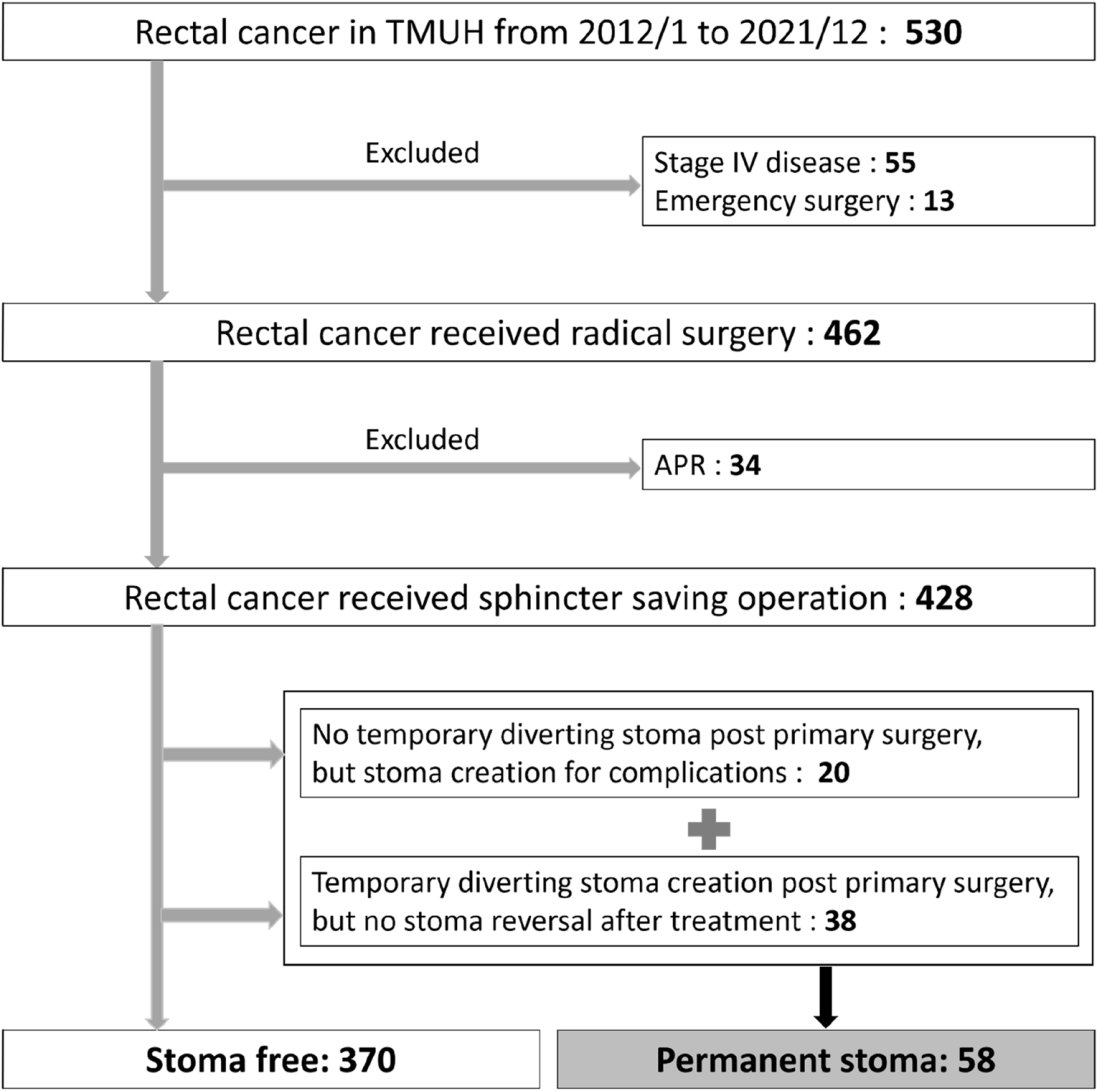
Patient selection flowchart. *APR* abdominoperineal resection, *TMUH* Taipei Medical University Hospital.

There were 530 patients diagnosed rectal cancer in Taipei Medical University Hospital from January 1, 2012, to December 31, 2021. We excluded 55 stage IV disease cases, 13 emergency surgery cases and 34 APR cases (Fig. 1). A total of 428 patients were included in this study, and their data were used in the training set. After a median follow-up of 59.3 (range 12–132) months, 58 (13.6%) of the 428 patients were confirmed to have a PS (Fig. 1).

Table 1 lists the baseline characteristics of all patients. We noted that 59.5% of the patients in the stoma-free group were men, and 55.2% of the patients in the PS group were women. The distance of the lesion from the anal verge considerably differed between the stoma-free and PS groups (7.0 vs 4.5 cm). A higher proportion of the patients in the PS received neoadjuvant CCRT (82.8% vs 73.0%). In the training dataset, over 60% of the patients had stage III disease.

**Table 1:** Characteristics of patients.

Variable	Overall (N = 428)	Stoma-free (n = 370)	Permanent stoma (n = 58)	p
Age, years	60.8 (± 12.9)	61.0 (± 12.8)	59.4 (± 13.6)	0.426
Sex				0.620
Male	246 (57.5%)	220 (59.6%)	26 (44.8%)	
Female	182 (42.5%)	150 (40.5%)	32 (55.2%)	
BMI, kg/m^2^	24.0 (± 4.0)	24.1 (± 4.1)	23.8 (± 3.7)	0.749
DM				0.297
Yes	87 (20.3%)	78 (21.1%)	9 (15.5%)	
No	341 (79.7%)	292 (78.9%)	49 (84.5%)	
Hypertension				0.611
Yes	140 (32.7%)	120 (32.4%)	20 (34.5%)	
No	288 (67.3%)	250 (67.6%)	38 (65.5%)	
Heart disease				0.623
Yes	36 (8.4%)	32 (8.6%)	4 (6.9%)	
No	392 (91.6%)	338 (91.4%)	54 (93.1%)	
COPD				0.087
Yes	5 (1.2%)	3 (0.8%)	2 (3.4%)	
No	423 (98.8%)	367 (99.2%)	56 (96.6%)	
CKD				0.973
Yes	43 (10.0%)	37 (10.0%)	6 (10.3%)	
No	385 (90.0%)	333 (90.0%)	52 (89.7%)	
Liver disease				0.914
Yes	49 (11.4%)	42 (11.4%)	7 (12.1%)	
No	379 (88.6%)	328 (88.6%)	51 (87.9%)	
Smoker				0.708
Yes	65 (15.2%)	57 (15.4%)	8 (13.8%)	
No	363 (84.8%)	313 (84.6%)	50 (86.2%)	
Distance to anus verge	6.7 (± 3.2)	7.0 (± 3.1)	4.5 (± 3.0)	< 0.001
Clinical T stage				0.239
T0–1	21 (4.9%)	20 (5.4%)	1 (1.7%)	
T2	61 (14.3%)	53 (14.3%)	8 (13.8%)	
T3	281 (65.7%)	245 (66.2%)	36 (62.1%)	
T4	32 (7.5%)	24 (6.5%)	8 (13.8%)	
Data loss	33 (7.7%)	28 (7.6%)	5 (8.6%)	
Clinical N stage				0.622
N0	125 (29.2%)	111 (30.0%)	14 (24.1%)	
N1	136 (31.8%)	116 (31.4%)	20 (34.5%)	
N2	134 (31.3%)	115 (31.1%)	19 (32.8%)	
Data loss	33 (7.1%)	28 (7.5%)	5 (8.6)	
AJCC c TNM stage				0.787
Stage 0–I	66 (15.4%)	59 (15.9%)	7 (12.1%)	
Stage II	60 (14.0%)	53 (14.3%)	7 (12.1%)	
Stage III	269 (62.9%)	230 (62.2%)	39 (67.2%)	
Data loss	33 (7.7%)	28 (7.6%)	5 (8.6%)	
Preoperative CCRT				0.098
Yes	318 (74.3%)	270 (73.0%)	48 (82.8%)	
No	110 (25.7%)	100 (27.0%)	10 (17.2%)	
Hb	12.8 (± 1.6)	12.8 (± 1.6)	12.7 (± 1.6)	0.591
Albumin	4.2 (± 0.4)	4.2 (± 0.4)	4.1 (± 0.4)	0.477
CEA	4.8 (± 8.2)	4.7 (± 8.2)	5.9 (± 7.6)	0.339
ASA score				0.086
I	29 (6.8%)	27 (7.3%)	2 (3.4%)	
II	351 (82.0%)	306 (82.7%)	45 (77.6%)	
III	48 (11.2%)	37 (10.0%)	11 (19.0%)	

### Model development and selection

**Figure 2:**
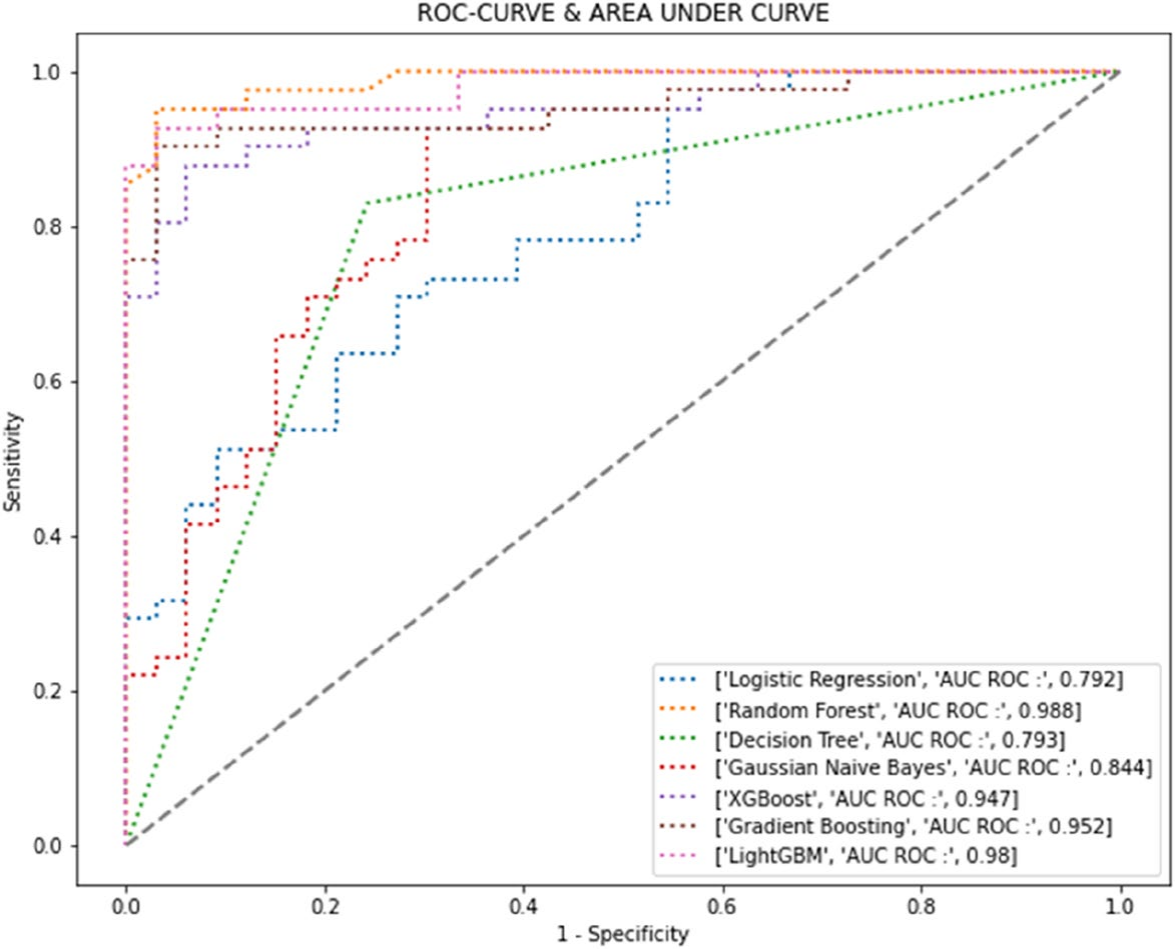
Area under the receiver operating characteristic for all candidate models used to predict the possibility of permanent stoma formation after sphincter-saving operation.

Seven machine learning models (LR, RF, DT, GNB, XGB, GB, and LGBM) were used to predict the probability of PS formation after a sphincter-saving operation. When these candidate prediction models were applied to the training dataset, a wide range of AUROC values (0.792–0.988) were obtained. The ROC curves are presented in Fig. 2. The accuracy, sensitivity, specificity, and AUROC of all candidate prediction models are listed in Table 2. Although RF appeared to be superior to other candidate models, with an accuracy of 0.953 and an AUROC of 0.988, we selected DT and LGBM. DT models decisions as tree-like structures that present each possible consequence and probability, and LGBM is based on a DT algorithm and used for ranking and classification.

**Table 2:** Accuracy, sensitivity, specificity, and area under the receiver operating characteristic for all candidate models used to predict the possibility of permanent stoma formation after sphincter-saving operation based on both the training dataset and testing dataset.

Dataset	Model	Accuracy	Sensitivity	Specificity	AUROC
Train dataset (n = 366)	Logistic regression	0.728	0.720	0.736	0.812
	Random forest	0.985	0.986	0.983	0.999
	Decision tree	0.976	0.976	0.976	0.976
	Gaussian Naïve Bayes	0.693	0.557	0.828	0.782
	Extreme gradient boosting	0.990	1.000	0.980	0.999
	Gradient boosting	0.973	0.983	0.963	0.996
	Light gradient Boosting machine	1.000	1.000	1.000	1.000
Testing dataset (n = 62)	Logistic regression	0.696	0.716	0.676	0.792
	Random forest	0.953	0.959	0.946	0.988
	Decision tree	0.791	0.757	0.824	0.793
	Gaussian Naïve Bayes	0.757	0.568	0.946	0.844
	Extreme gradient boosting	0.892	0.932	0.851	0.947
	Gradient boosting	0.932	0.959	0.905	0.952
	Light gradient boosting machine	0.926	0.905	0.946	0.980

### DT and clinical scenarios

**Figure 3:**
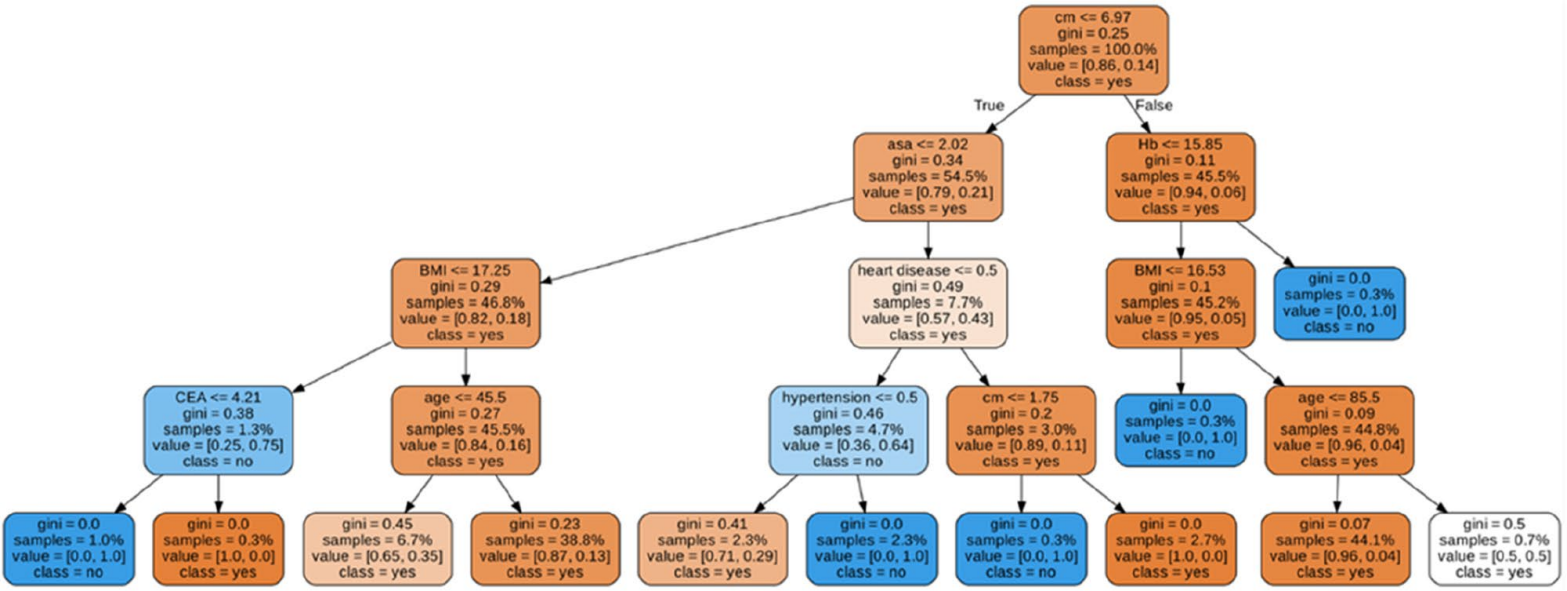
Decision tree for permanent stoma risk prediction.

A DT model was developed using the training dataset. The Gini index was used to select variables, and the final tree was pruned. Eight input variables remained in this model: the distance from the lesion to the anal verge, ASA score, age, BMI, presence of heart disease, presence of hypertension, preoperative Hb levels, and preoperative CEA levels. Figure 3 presents the final DT with a size of 10 nodes, 12 leaves, and 4 layers. In the DT model, the root node represented the distance from the lesion to the anal verge. This node split the sample population into 2 groups: those with a distance of < 7 cm from the lesion to the anal verge on the left and those with a distance of ≥ 7 cm from the lesion to the anal verge on the right. Other variables in the model served as decision conditions at the nodes to guide the model in making predictions. The DT model had an accuracy of 0.791, a sensitivity of 0.757, a specificity 0.824, and an AUROC of 0.793.

In Table 3, we present some examples of DT predictions for typical scenarios. For example, both patient 1 and patient 2 had low rectal cancer and an ASA score of 2. However, the higher BMI and older age of patient 1 markedly increased the probability of PS formation to 38.8%. Additionally, for patient 3, the distance from the lesion to the anal verge was longer than those of patients 1 and 2, which indicated a lower probability of PS formation in patient 3. In addition, patient 3 was younger and had a lower BMI and no comorbidities, which indicated a higher likelihood of this patient being stoma-free.

**Table 3:** Prediction examples for typical scenarios based on the decision tree model.

Decision tree layer	Chance node	Patient 1	Patient 2	Patient 3
Layer 1	Distance from the lesion to the anal verge, cm	5	5	10
Layer 2	ASA score	2	2	2
	Hb, g/dL	11.8	13.8	16.1
Layer 3	BMI, kg/m^2^	29.2	22.5	20.3
	Heart disease	No	No	No
Layer 4	Age, years	71	43	44
	Hypertension	No	No	No
	CEA, ng/mL	1.4	1.6	0.5
Stoma risk in percent		38.8%	6.7%	0.3%

### LGBM for ranking

**Figure 4:**
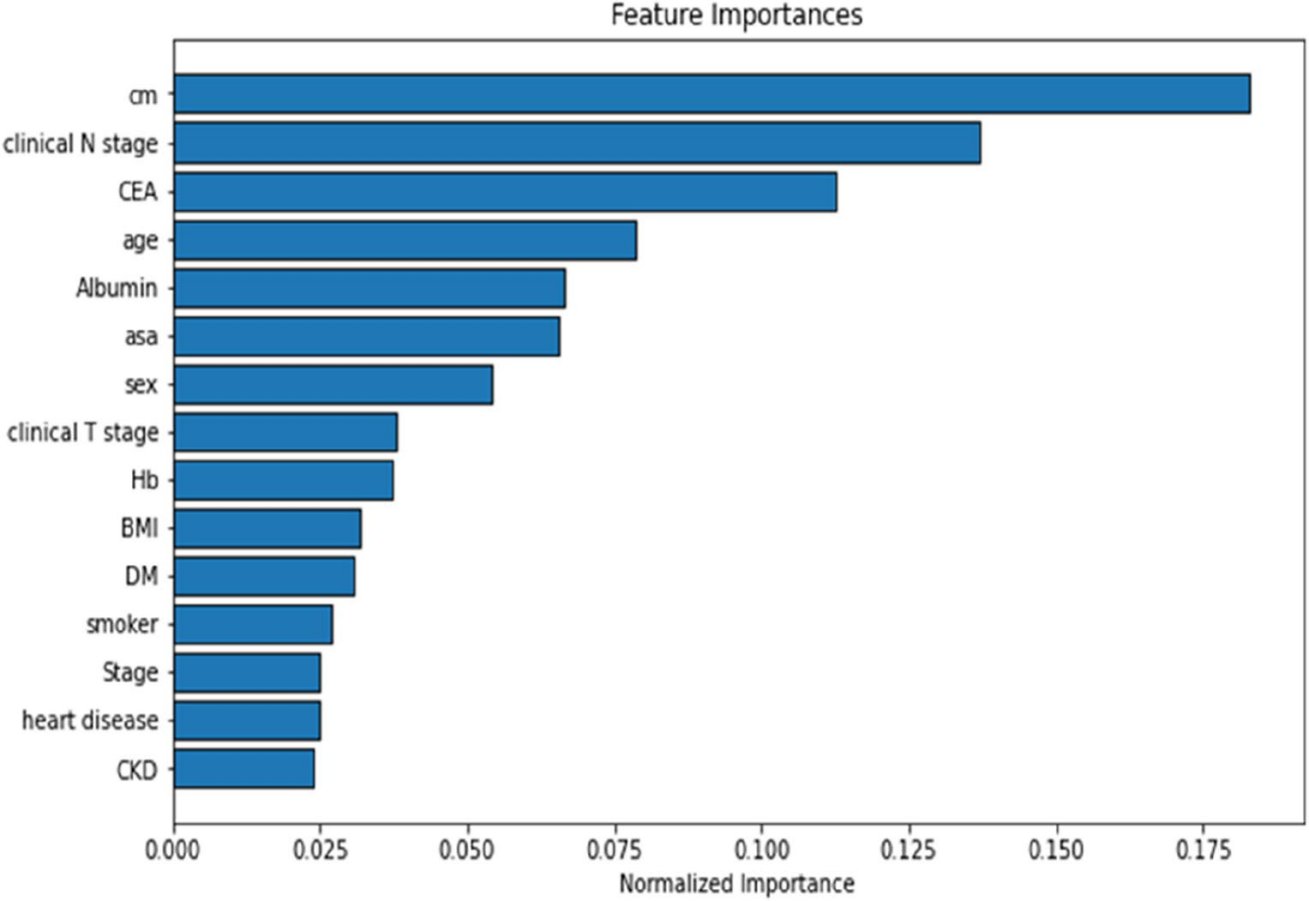
Normalized importance of risk factors for permanent stoma determined using light gradient boosting machine model.

According to our normalization of the LGBM model presented in Fig. 4, the distance from the lesion to the anal verge had the highest impact index, followed by clinical N stage, age, sex, ASA score, preoperative albumin levels, and preoperative CEA levels. Smoking status and presence of comorbidities did not exert a considerable effect on PS formation. The LGBM model had an accuracy of 0.926, a sensitivity of 0.905, a specificity of 0.946, and an AUROC of 0.980.

## Discussion

Treatment for rectal cancer is complex and difficult despite advancements having been made in medical therapy, surgical techniques, and instrumentation. For patients with rectal cancer who are suitable for sphincter-saving operations, the restoration of bowel continuity after treatment remains a challenge because 3–25% of such patients develop a PS^5,6^. The formation of a PS is usually beyond the expectations of physicians and patients and considerably reduces the quality of life of patients. The presence of a stoma was identified as a key factor deteriorating the quality of life of patients with colorectal cancer^7^. Patients living with a stoma may encounter stoma-related complications and technical problems, such as bulges or hernias, uncontrollable gas and fecal passage, odor, peristomal skin irritation, and leakage around the stoma or appliance^7,8^. Moreover, some problems, including changes in body image, psychological distress or anxiety, stigma, embarrassment, and social isolation, may affect daily normal activities^7–9^. Therefore, the possibility of PS formation should be discussed when surgical informed consent is obtained before rectal cancer surgery, even if it was not initially planned^10^. The goal of obtaining surgical informed consent is to enhance patients’ understanding of the intended procedure, increase patient satisfaction, maintain trust between patients and health providers, and minimize litigation issues involving surgical procedures^3^. However, the pathophysiological mechanism underlying the process of anastomotic regeneration remains poorly understood, which increases the difficulty of predicting the occurrence of anastomotic complications^11^, despite some risk factors being well-known. Researchers investigated the risk factors for and the causes of PS formation^12^. However, few studies have precisely predicted the probability of PS formation before surgery. In the current study, we used AI to define a procedure-specific core information set for predicting and calculating PS probability. This information can be used in the process of obtaining preoperative informed consent.

AI has been increasingly applied because of improvements in computer power and technology. AI models can process large amounts of data and can extract information from complex medical images that may be difficult to detect through human analysis alone^13^. Deep learning, an algorithm that uses artificial neural networks to perform representation learning on data, has been applied in numerous areas of health care, including diagnostic imaging analysis, pathological interpretation, disease prognosis prediction, genetic analysis, simulation of physiological conditions, and drug design^2,14^. In some cases, AI models have exhibited performance comparable to that of human experts. In the field of colorectal cancer, Bychkov et al.^15^ reported that deep learning achieved an expert-level accuracy for pathological interpretation. In addition, deep learning models based on magnetic resonance imaging and radiomics data have exhibited satisfactory performance in predicting the pathological complete response of patients with locally advanced rectal cancer after neoadjuvant CCRT and have thus provided a diagnostic reference for clinicians^16,17^. AI models have also been used to predict the possibility of postoperative leakage^18^, lymph node metastasis^19^, distant metastasis^20^, and disease survival^21^. These predictive results may affect clinicians’ decisions, lead to changes in disease therapy, and aid in developing new treatment methods. For example, Ichimasa et al.^22^ reported that AI significantly reduced the occurrence of unnecessary additional surgery after the endoscopic resection of T1 colorectal cancer without missing lymph node metastasis positivity. Most analytical and predictive methods used to analyze patients with rectal cancer after sphincter-saving operations have been based on Cox proportional hazard regression, Kaplan–Meier analysis, and log-rank tests. Back et al.^10^ developed an AI-based model to predict the probability of PS. They determined that GB and Naïve Bayes were the most satisfactory models, with an estimated AUROC of 0.68 (0.63–0.72). However, their study used registry data. Some potentially crucial predictors, including patient comorbidities, CEA levels, nutritional status, and smoking status, were not included in the AI analysis.

In the current study, we included several routinely available preoperative data in our analysis. These data included smoking history, comorbidities, and preoperative laboratory data. We used 7 prediction models and selected the DT and LightGBM models. Random forests generally outperform decision trees for several reasons: They effectively address the issue of overfitting by aggregating the outputs of multiple decision trees to formulate a final prediction. The DT is a tree-like model of decisions that presents each possible consequence and probability, and the LightGBM model is based on DT algorithms and used for ranking and classification. The accuracies of the RF, DT and LightGBM models were 95%, 79% and 93%, respectively. The AUROC values of the RF, DT and LightGBM models were 0.99, 0.79 and 0.98, respectively. All models exhibited excellent prediction ability. The DT algorithm is widely used in medicine because it enables the comprehensive representation of a model. A decision tree integrates certain choices, while a random forest merges multiple decision trees. Consequently, this constitutes a lengthier albeit slower procedure. Conversely, a decision tree is swift and functions smoothly with extensive datasets, particularly those of linear nature. Rigorous training is essential for the random forest model. Decision tree is also known for its interpretability as one of the explainable AI algorithm (XAI). The allocation rule of a DT can be easily visualized by plotting the tree structure. Most importantly, Standard computational resources are sufficient for the incorporation of tree-based models into electronic decision support systems and thus, improve shared decision making process in clinical practice. Our DT model revealed that a shorter distance from the lesion to the anal verge, older age, and higher BMI were risk factors for PS formation. Therefore, patients with these risk factors should be informed of the risk of PS formation when informed consent is obtained from them prior to an operation. The LightGBM model is a GB framework that uses tree-based learning algorithms^23^. The LightGBM algorithm can effectively handle the relationships, distributions, and ranking of data. According to our LightGBM model, the key risk factors for PS, in descending order, were the distance of the lesion from the anal verge, clinical N stage, age, sex, ASA score, and preoperative albumin and CEA levels.

In patients with rectal cancer, the distance of the lesion from the anal verge is among the most crucial factors affecting the feasibility of a sphincter-saving operation. The most challenging aspect of rectal cancer surgery is ensuring oncologically safe resection within the narrow pelvis while preserving the anus and sphincter complex^24^. The anastomosis being in a lower position increases the risk of complications, such as anastomotic leak, stricture, and anorectal incontinence^25^. Anastomotic leak is the main reason for PS formation. Lindgren et al.^26^ and Jutesten et al.^27^ have reported that over 56–65% of patients with symptomatic anastomotic leak develop a PS. Clinical N stage positivity indicates a more advanced cancer stage, with stage III being the minimum. Patients with cancer at such a stage have a higher chance of developing local recurrence, which is another reason for PS formation because it causes mechanical bowel obstruction^28^. Neoadjuvant CCRT is required for such patients to reduce the recurrence rate. However, some studies have identified neoadjuvant CCRT as a common risk factor for PS formation^10,27^. Tabchouri et al.^29^ reported that patients with rectal cancer receiving neoadjuvant CCRT had higher incidence rates of postoperative anastomotic leakage and sepsis. In addition, neoadjuvant radiation has been increasingly associated with postoperative bowel dysfunction, including low anterior resection syndrome^30^. Some postoperative patients with poor sphincter function may have a higher risk of PS formation. Third, CEA is a complex glycoprotein produced by 90% of colorectal cancers^31^. Since the 1970s, postoperative measurement of the CEA level has been included in the follow-up surveillance procedure for patients with colorectal cancer. Early increased postoperative CEA levels are often a sign of remaining cancer tissues, whereas a later increase indicates cancer recurrence^32^. Preoperative CEA has been identified as a predictor of recurrence, resectability, and survival after the resection of colorectal cancer^32,33^. A higher preoperative CEA level is associated with a higher local recurrence rate and an increased probability of PS formation.

### Limitations

This study has some limitations that should be addressed. First, all patients were included from a single center. Although we included more than 400 patients in this analysis, the amount of patient data remained inadequate. Inclusion of a large amount of data from more patients would increase the accuracy of the AI model. Therefore, data should be collected from multiple centers to develop benchmark databases. Second, this study had a retrospective design, and several forms of data were missing from the collected medical records. Missing data can reduce the predictive power of an AI model. Third, information on other, more specific clinical items should be incorporated to improve the predictive ability of the AI model. Such information may include socioeconomic class, medication use, sphincter function, and tumor histological characteristics determined in preoperative colonoscopy biopsy. Fourth, patient data are often imbalanced. Using imbalanced data in model training can reduce the model performance. Under-sampling the majority group can be used to solve this problem. However, this solution is suboptimal. Another solution to this problem is the generation of synthetic data. Fifth, external validation was not performed. Data from other centers are required to verify the discriminatory ability of this model. Finally, the internal mechanisms of AI analysis remain difficult to understand. Collaboration between researchers with expertise in biomedical research and machine learning is necessary to improve the performance of AI models.

## Conclusions

The present study’s integration of AI with clinically available preoperative data demonstrated potential for use in predicting PS formation after a sphincter-saving operation. Our model exhibited excellent predictive ability and can improve the process of obtaining informed consent from patients. In the future, we will optimize the system by collecting more clinical data and adding data from other centers.

### Supplementary Information

Supplementary Figure 1.

Supplementary Table 1.

## Data Availability

The data that support the findings of this study are available from the corresponding author upon reasonable request.

## References

[1] Jiang F, Jiang Y, Zhi H, Dong Y, Li H, Ma S, Wang Y, Dong Q, Shen H, Wang Y (2017). Artificial intelligence in healthcare: Past, present, and future. Stroke Vasc. Neurol..

[2] Elemento O, Leslie C, Lundin J, Tourassi G (2021). Artificial intelligence in cancer research, diagnosis, and therapy. Nat. Rev. Cancer..

[3] Teshome M, Wolde Z, Gedefaw A, Tariku M, Asefa A (2018). Surgical informed consent in obstetric and gynecologic surgeries: Experience from a comprehensive teaching hospital in Southern Ethiopia. BMC Med. Ethics..

[4] Scheer AS, O'Connor AM, Chan BP, Moloo H, Poulin EC, Mamazza J, Auer RC, Boushey RP (2012). The myth of informed consent in rectal cancer surgery. Dis. Colon Rectum..

[5] Kuo CY, Lin YK, Wei PL, Chi-Yong Ngu J, Lee KD, Chen CL, Huang Y, Chen CC, Kuo LJ (2022). Clinical assessment for non-reversal stoma and stoma re-creation after reversal surgery for rectal cancer patients after sphincter-saving operation. Asian J. Surg..

[6] Holmgren K, Kverneng Hultberg D, Haapamäki MM, Matthiessen P, Rutegård J, Rutegård M (2017). High stoma prevalence and stoma reversal complications following anterior resection for rectal cancer: A population based multicenter study. Colorectal Dis..

[7] Näsvall P, Dahlstrand U, Löwenmark T, Rutegård J, Gunnarsson U, Strigård K (2017). Quality of life in patients with a permanent stoma after rectal cancer surgery. Qual. Life Res..

[8] Richbourg L, Thorpe JM, Rapp CG (2007). Difficulties experienced by the ostomate after hospital discharge. J. Wound Ostomy Cont. Nurs..

[9] Cakmak A, Aylaz G, Kuzu MA (2010). Permanent stoma not only affects patients’ quality of life but also that of their spouses. World J. Surg..

[10] Back E, Häggström J, Holmgren K, Haapamäki MM, Matthiessen P, Rutegård J, Rutegård M (2021). Permanent stoma rates after anterior resection for rectal cancer: Risk prediction scoring using preoperative variables. Br. J. Surg..

[11] Guyton KL, Hyman NH, Alverdy JC (2016). Prevention of perioperative anastomotic healing complications: Anastomotic stricture and anastomotic leak. Adv. Surg..

[12] Zhou X, Wang B, Li F, Wang J, Fu W (2017). Risk factors associated with nonclosure of defunctioning stomas after sphincter-preserving low anterior resection of rectal cancer: A meta-analysis. Dis. Colon Rectum..

[13] Levine AB, Schlosser C, Grewal J, Coope R, Jones SJM, Yip S (2019). Rise of the machines: Advances in deep learning for cancer diagnosis. Trends Cancer..

[14] Zhu W, Xie L, Han J, Guo X (2020). The application of deep learning in cancer prognosis prediction. Cancers (Basel)..

[15] Bychkov D, Linder N, Turkki R, Nordling S, Kovanen PE, Verrill C, Walliander M, Lundin M, Haglund C, Lundin J (2018). Deep learning-based tissue analysis predicts outcome in colorectal cancer. Sci. Rep..

[16] Zhang XY, Wang L, Zhu HT, Li ZW, Ye M, Li XT, Shi YJ, Zhu HC, Sun YS (2020). Predicting rectal cancer response to neoadjuvant chemoradiotherapy using deep learning of diffusion kurtosis MRI. Radiology..

[17] Bibault JE, Giraud P, Housset M, Durdux C, Taieb J, Berger A, Coriat R, Chaussade S, Dousset B, Nordlinger B, Burgun A (2018). Deep learning and radiomics predict complete response after neo-adjuvant chemoradiation for locally advanced rectal cancer. Sci. Rep..

[18] Mazaki J, Katsumata K, Ohno Y, Udo R, Tago T, Kasahara K, Kuwabara H, Enomoto M, Ishizaki T, Nagakawa Y, Tsuchida A (2021). A novel predictive model for anastomotic leakage in colorectal cancer using auto-artificial intelligence. Anticancer Res..

[19] Zhao X, Xie P, Wang M, Li W, Pickhardt PJ, Xia W, Xiong F, Zhang R, Xie Y, Jian J, Bai H, Ni C, Gu J, Yu T, Tang Y, Gao X, Meng X (2020). Deep learning-based fully automated detection and segmentation of lymph nodes on multiparametric-mri for rectal cancer: A multicentre study. EBioMedicine..

[20] Liu X, Zhang D, Liu Z, Li Z, Xie P, Sun K, Wei W, Dai W, Tang Z, Ding Y, Cai G, Tong T, Meng X, Tian J (2021). Deep learning radiomics-based prediction of distant metastasis in patients with locally advanced rectal cancer after neoadjuvant chemoradiotherapy: A multicentre study. EBioMedicine..

[21] Yu H, Huang T, Feng B, Lyu J (2022). Deep-learning model for predicting the survival of rectal adenocarcinoma patients based on a surveillance, epidemiology, and end results analysis. BMC Cancer..

[22] Ichimasa K, Kudo SE, Mori Y, Misawa M, Matsudaira S, Kouyama Y, Baba T, Hidaka E, Wakamura K, Hayashi T, Kudo T, Ishigaki T, Yagawa Y, Nakamura H, Takeda K, Haji A, Hamatani S, Mori K, Ishida F, Miyachi H (2018). Artificial intelligence may help in predicting the need for additional surgery after endoscopic resection of T1 colorectal cancer. Endoscopy..

[23] Rufo DD, Debelee TG, Ibenthal A, Negera WG (2021). Diagnosis of diabetes mellitus using gradient boosting machine (LightGBM). Diagnostics (Basel)..

[24] Piozzi GN, Baek SJ, Kwak JM, Kim J, Kim SH (2021). Anus-preserving surgery in advanced low-lying rectal cancer: A perspective on oncological safety of intersphincteric resection. Cancers (Basel)..

[25] Junginger T, Gönner U, Trinh TT, Lollert A, Oberholzer K, Berres M (2010). Permanent stoma after low anterior resection for rectal cancer. Dis. Colon Rectum..

[26] Lindgren R, Hallböök O, Rutegård J, Sjödahl R, Matthiessen P (2011). What is the risk for a permanent stoma after low anterior resection of the rectum for cancer? A six-year follow-up of a multicenter trial. Dis. Colon Rectum..

[27] Jutesten H, Draus J, Frey J, Neovius G, Lindmark G, Buchwald P, Lydrup ML (2019). High risk of permanent stoma after anastomotic leakage in anterior resection for rectal cancer. Colorectal Dis..

[28] Bouchard P, Efron J (2010). Management of recurrent rectal cancer. Ann. Surg. Oncol..

[29] Tabchouri N, Eid Y, Manceau G, Frontali A, Lakkis Z, Salame E, Lecomte T, Chapet S, Calais G, Heyd B, Karoui M, Alves A, Panis Y, Ouaissi M (2020). Neoadjuvant treatment in upper rectal cancer does not improve oncologic outcomes but increases postoperative morbidity. Anticancer Res..

[30] Li X, Fu R, Ni H, Du N, Wei M, Zhang M, Shi Y, He Y, Du L (2023). Effect of neoadjuvant therapy on the functional outcome of patients with rectal cancer: A systematic review and meta-analysis. Clin. Oncol. (R. Coll. Radiol.)..

[31] Wiggers T, Arends JW, Schutte B, Volovics L, Bosman FT (1988). A multivariate analysis of pathologic prognostic indicators in large bowel cancer. Cancer..

[32] Tarantino I, Warschkow R, Worni M, Merati-Kashani K, Köberle D, Schmied BM, Müller SA, Steffen T, Cerny T, Güller U (2012). Elevated preoperative CEA is associated with worse survival in stage I–III rectal cancer patients. Br. J. Cancer..

[33] Bhatti I, Patel M, Dennison AR, Thomas MW, Garcea G (2015). Utility of postoperative CEA for surveillance of recurrence after resection of primary colorectal cancer. Int. J. Surg..

