# Supplementary figures and images for: Artificial intelligence based system for predicting permanent stoma after sphincter saving operations
Source: Sci Rep. 2023 Sep 25;13:16039. doi: 10.1038/s41598-023-43211-w (PMC10519982; doi:10.1038/s41598-023-43211-w)

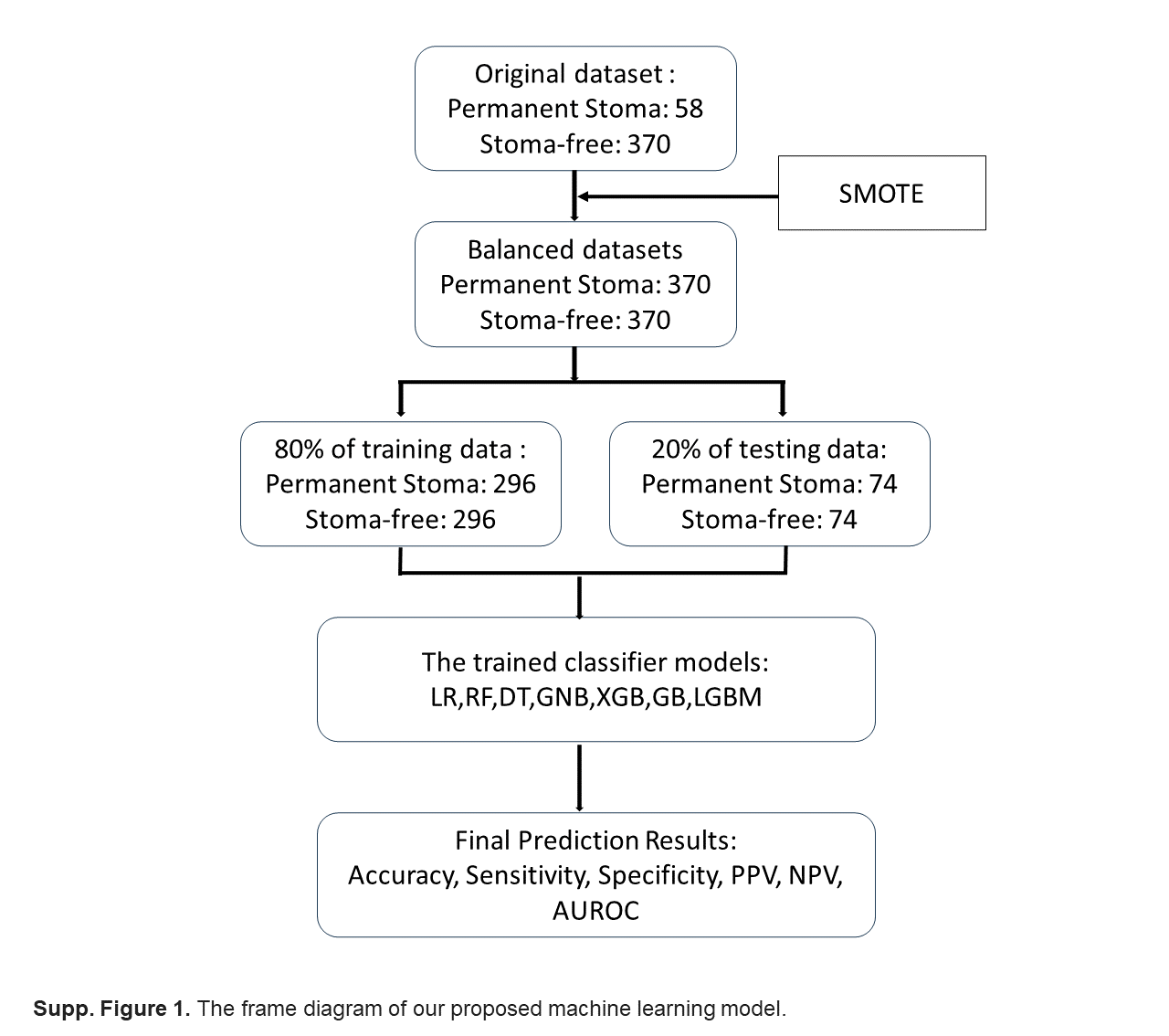

Supplement: Supplementary file 1 — Supplementary Figure 1. [file 41598_2023_43211_MOESM1_ESM.png]
