# Supplementary material for: Artificial intelligence based system for predicting permanent stoma after sphincter saving operations
Source: Sci Rep. 2023 Sep 25;13:16039. doi: 10.1038/s41598-023-43211-w (PMC10519982; doi:10.1038/s41598-023-43211-w)
Supplement: Supplementary file 2 — Supplementary Table 1. [file 41598_2023_43211_MOESM2_ESM.docx]

Supplementary Table 1. Confusion Matrix after SMOTE algorithm

|  |  |  | Predicted:  stoma | Predicted:  stoma-free | Accuracy | Sensitivity | Specificity | PPV | NPV | TP | FN | FP | TN |
| --- | --- | --- | --- | --- | --- | --- | --- | --- | --- | --- | --- | --- | --- |
| Training | Logistic Regression | Ground truth : stoma | 213 | 83 | 0.728 | 0.720 | 0.736 | 0.732 | 0.724 | 213 | 83 | 78 | 218 |
|  |  | Ground truth : stoma-free | 78 | 218 |  |  |  |  |  |  |  |  |  |
|  | Random Forest | Ground truth : stoma | 292 | 4 | 0.985 | 0.986 | 0.983 | 0.983 | 0.986 | 292 | 4 | 5 | 291 |
|  |  | Ground truth : stoma-free | 5 | 291 |  |  |  |  |  |  |  |  |  |
|  | Decision Tree | Ground truth : stoma | 289 | 7 | 0.976 | 0.976 | 0.976 | 0.976 | 0.976 | 289 | 7 | 7 | 289 |
|  |  | Ground truth : stoma-free | 7 | 289 |  |  |  |  |  |  |  |  |  |
|  | Gaussian Naïve Bayes | Ground truth : stoma | 165 | 131 | 0.693 | 0.557 | 0.828 | 0.764 | 0.652 | 165 | 131 | 51 | 245 |
|  |  | Ground truth : stoma-free | 51 | 245 |  |  |  |  |  |  |  |  |  |
|  | Extreme Gradient Boosting | Ground truth : stoma | 296 | 0 | 0.990 | 1.000 | 0.980 | 0.980 | 1.000 | 296 | 0 | 6 | 290 |
|  |  | Ground truth : stoma-free | 6 | 290 |  |  |  |  |  |  |  |  |  |
|  | Gradient Boosting | Ground truth : stoma | 291 | 5 | 0.973 | 0.983 | 0.963 | 0.964 | 0.983 | 291 | 5 | 11 | 285 |
|  |  | Ground truth : stoma-free | 11 | 285 |  |  |  |  |  |  |  |  |  |
|  | Light Gradient Boosting Machine | Ground truth : stoma | 296 | 0 | 1.000 | 1.000 | 1.000 | 1.000 | 1.000 | 296 | 0 | 0 | 296 |
|  |  | Ground truth : stoma-free | 0 | 296 |  |  |  |  |  |  |  |  |  |

|  |  |  | Predicted:  stoma | Predicted:  stoma-free | Accuracy | Sensitivity | Specificity | PPV | NPV | TP | FN | FP | TN |
| --- | --- | --- | --- | --- | --- | --- | --- | --- | --- | --- | --- | --- | --- |
| Testing | Logistic Regression | Ground truth : stoma | 53 | 21 | 0.696 | 0.716 | 0.676 | 0.688 | 0.704 | 53 | 21 | 24 | 50 |
|  |  | Ground truth : stoma-free | 24 | 50 |  |  |  |  |  |  |  |  |  |
|  | Random Forest | Ground truth : stoma | 71 | 3 | 0.953 | 0.959 | 0.946 | 0.947 | 0.959 | 71 | 3 | 4 | 70 |
|  |  | Ground truth : stoma-free | 4 | 70 |  |  |  |  |  |  |  |  |  |
|  | Decision Tree | Ground truth : stoma | 56 | 18 | 0.791 | 0.757 | 0.824 | 0.812 | 0.772 | 56 | 18 | 13 | 61 |
|  |  | Ground truth : stoma-free | 13 | 61 |  |  |  |  |  |  |  |  |  |
|  | Gaussian Naïve Bayes | Ground truth : stoma | 42 | 32 | 0.757 | 0.568 | 0.946 | 0.913 | 0.686 | 42 | 32 | 4 | 70 |
|  |  | Ground truth : stoma-free | 4 | 70 |  |  |  |  |  |  |  |  |  |
|  | Extreme Gradient Boosting | Ground truth : stoma | 69 | 5 | 0.892 | 0.932 | 0.851 | 0.863 | 0.926 | 69 | 5 | 11 | 63 |
|  |  | Ground truth : stoma-free | 11 | 63 |  |  |  |  |  |  |  |  |  |
|  | Gradient Boosting | Ground truth : stoma | 71 | 3 | 0.932 | 0.959 | 0.905 | 0.910 | 0.957 | 71 | 3 | 7 | 67 |
|  |  | Ground truth : stoma-free | 7 | 67 |  |  |  |  |  |  |  |  |  |
|  | Light Gradient Boosting Machine | Ground truth : stoma | 67 | 7 | 0.926 | 0.905 | 0.946 | 0.944 | 0.909 | 67 | 7 | 4 | 70 |
|  |  | Ground truth : stoma-free | 4 | 70 |  |  |  |  |  |  |  |  |  |

<Note>PPV: Positive Predictive Value; NPV: Negative Predictive Value; TP: True Positive; FN: False Negative; FP: False Positive; TN: True Negative
